# MicroCT reveals domesticated rice (*Oryza sativa*) within pottery sherds from early Neolithic sites (4150–3265 cal BP) in Southeast Asia

**DOI:** 10.1038/s41598-017-04338-9

**Published:** 2017-08-07

**Authors:** Aleese Barron, Michael Turner, Levi Beeching, Peter Bellwood, Philip Piper, Elle Grono, Rebecca Jones, Marc Oxenham, Nguyen Khanh Trung Kien, Tim Senden, Tim Denham

**Affiliations:** 1 School of Archaeology and Anthropology, Australian National University, Canberra, ACT 2601 Australia; 2 National Laboratory for X-ray Computed Tomography, Australian National University, Canberra, ACT 2601 Australia; 3 Centre for Archaeological Studies, Southern Institute for Social Sciences, Ho Chi Minh City, Vietnam; 4 Research School of Physics and Engineering, Australian National University, Canberra, ACT 2601 Australia

## Abstract

Rice (*Oryza sativa*) was domesticated in the Yangtze Valley region at least 6000–8000 years ago, yet the timing of dispersal of domesticated rice to Southeast Asia is contentious. Often rice is not well-preserved in archaeobotanical assemblages at early Neolithic sites in the wet tropics of Southeast Asia and consequently rice impressions in pottery have been used as a proxy for rice cultivation despite their uncertain taxonomic and domestication status. In this research, we use microCT technology to determine the 3D microscale morphology of rice husk and spikelet base inclusions within pottery sherds from early Neolithic sites in Vietnam. In contrast to surface impressions, microCT provides images of the entire husk and spikelet base preserved within the pottery, including the abscission scar characteristic of domesticated rice. This research demonstrates the potential of microCT to be a new, non-destructive method for the identification of domesticated plant remains within pottery sherds, especially in contexts where archaeobotanical preservation is poor and chaff-tempered sherds are rare and unavailable for destructive analysis. The method has the potential to greatly advance the understanding of crop domestication and agricultural dispersal for ceramic cultures in different parts of the world.

## Introduction

During the domestication process rice (*Oryza sativa*), like other cereals, developed a non-shattering spikelet base to enable harvesting^1, 2^. The domesticated spikelet base has a diagnostic, vertically asymmetric, deeply recessed abscission scar visible in archaeobotanical assemblages that enables discrimination from wild rice^3, 4^. Rice was domesticated in the Yangtze River Valley region of China at least 6000–8000 years ago^5, 6^, yet the timing of dispersal of domesticated rice to Southeast Asia and its cultural associations are contentious^7, 8^.

The earliest domesticated rice (*Oryza sativa*) in Southeast Asia spread southward from its source region in China after 7000 cal BP potentially along with other Neolithic traits, such as cultivation practices, pottery and domesticated pigs, reaching Guangdong, Hainan and Taiwan by about 5500–5000 cal BP^9–11^. Archaeobotanical evidence for domesticated rice at early Neolithic sites in Southeast Asia is sparse, largely due to the poor preservation of macrobotanical remains in wet tropical climates and the inconsistent application of retrieval methods, as well as the uncertain taxonomic and domesticated status of microfossils^12–14^. Consequently, archaeological studies in the region have used rice impressions in pottery as a proxy for rice cultivation despite their uncertain taxonomic and domestication status^12–14^. This study sought to develop a new method to identify and determine the domestication status of rice remains within pottery efficiently and non-destructively.

The primary diagnostic criterion for the accurate discrimination of domesticated rice is the deeply recessed and asymmetric abscission scar at the base of spikelets characteristic of non-shattering cultivars^3–6^. MicroCT (where voxel size can be 1 µm or smaller) is a non-destructive method able to provide the 3D morphological and contextual detail to identify and visualise individual rice husks, spikelet bases and abscission scars for included organic material and impressions within pottery. In this study, pottery sherds from three archaeological sites in southern Vietnam were subjected to microCT analysis to determine if organic inclusions, or combusted impressions of organic inclusions, of domesticated rice (*Oryza sativa*) were present.

**Figure 1:**
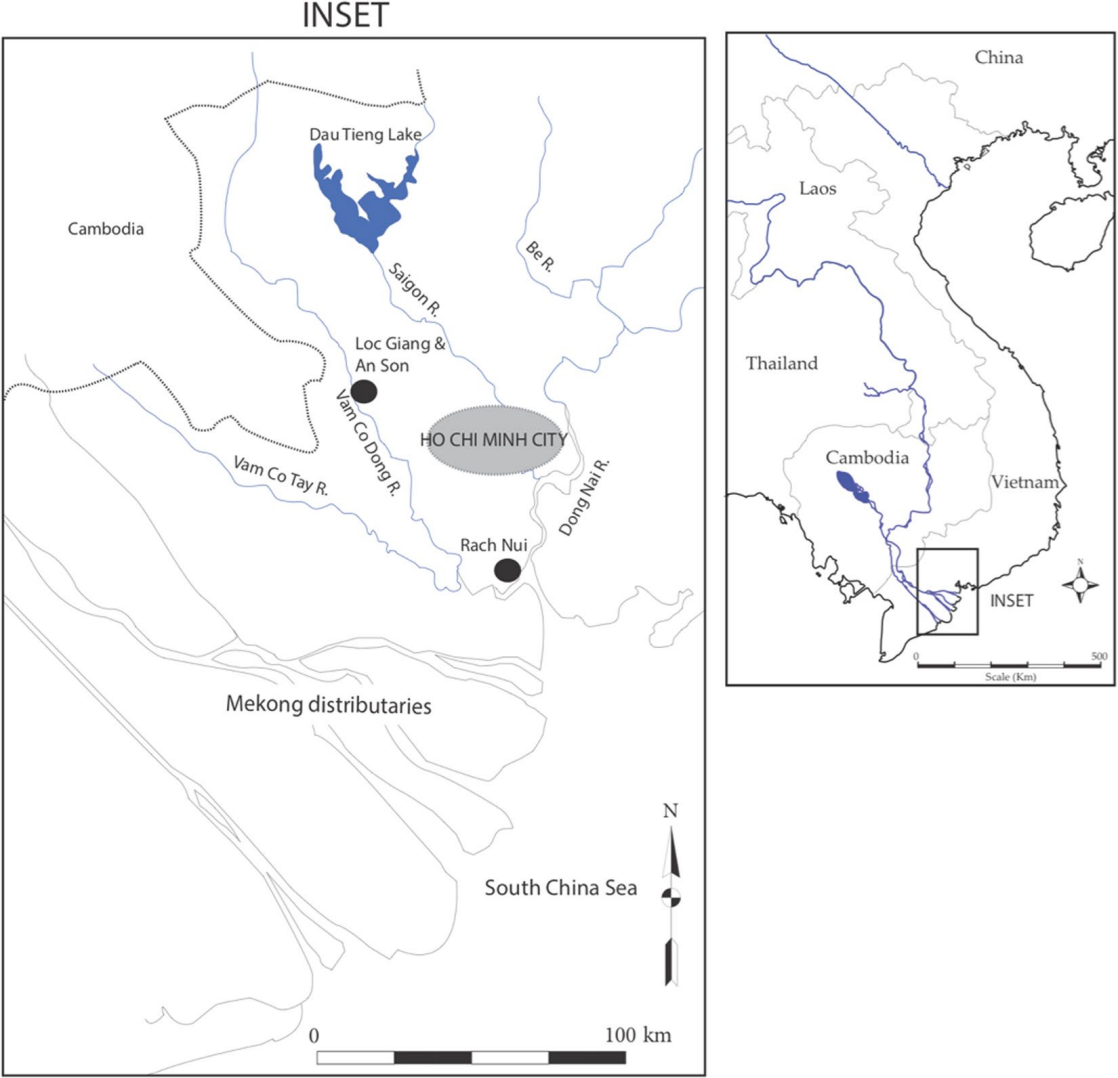
Map of Vietnam (right) with inset showing location of sites (left). Map created by Bellwood, P. & Piper, P. using Adobe Illustrator version CS5.

The archaeological sites date to the southern Vietnam Neolithic (Fig. 1): An Son to c.4200–3150 cal BP^15^, Loc Giang from at least 4000–3300 cal BP^16^ and Rach Nui to 3555–3265 cal BP^17^. Each site is an artificial mound averaging one hectare in extent, which rises 4–6 m above the surrounding alluvial or estuarine landscape, and contains artificially-laid floors with post-holes representing former timber constructions^15–20^. Habitation layers contain bones of domesticated pigs and dogs, together with decorated pottery, stone adzes and bone tools. An Son and Loc Giang have riverine locations, whereas Rach Nui is located in a mangrove-flanked estuary.

An Son and Loc Giang are currently the oldest excavated Neolithic sites in the greater Mekong region of southern Vietnam, dating to between c.4200 and 3200 cal BP. The stamped and incised pottery suggests widespread relationships with other regions of the Greater Mekong Basin, especially in Thailand, and ultimately with southern China^18–20^. Rach Nui is a slightly younger site, with cultural materials sufficiently different from those of An Son and Loc Giang (which have identical material cultures) to suggest occupation by a group with a different ethnic and economic orientation.

Sparse and often indeterminate evidence of rice has previously been reported for archaeobotanical and pottery assemblages from An Son, Loc Giang and Rach Nui. At An Son, a single rice husk was extracted from a poorly fired pottery sherd, which genetic analysis suggests is the domesticated species *Oryza sativa* ssp. *japonica* ^15^. At Loc Giang, archaeobotanical research identified one domestic-type spikelet base, as well as multiple lines of evidence for rice of indeterminate domestication status, including husk impressions in pottery, two phytoliths and a charred rice husk. At Rach Nui, two domesticated spikelet bases and indeterminate rice husks were extracted from sediment samples^17, 21^.

**Figure 2:**
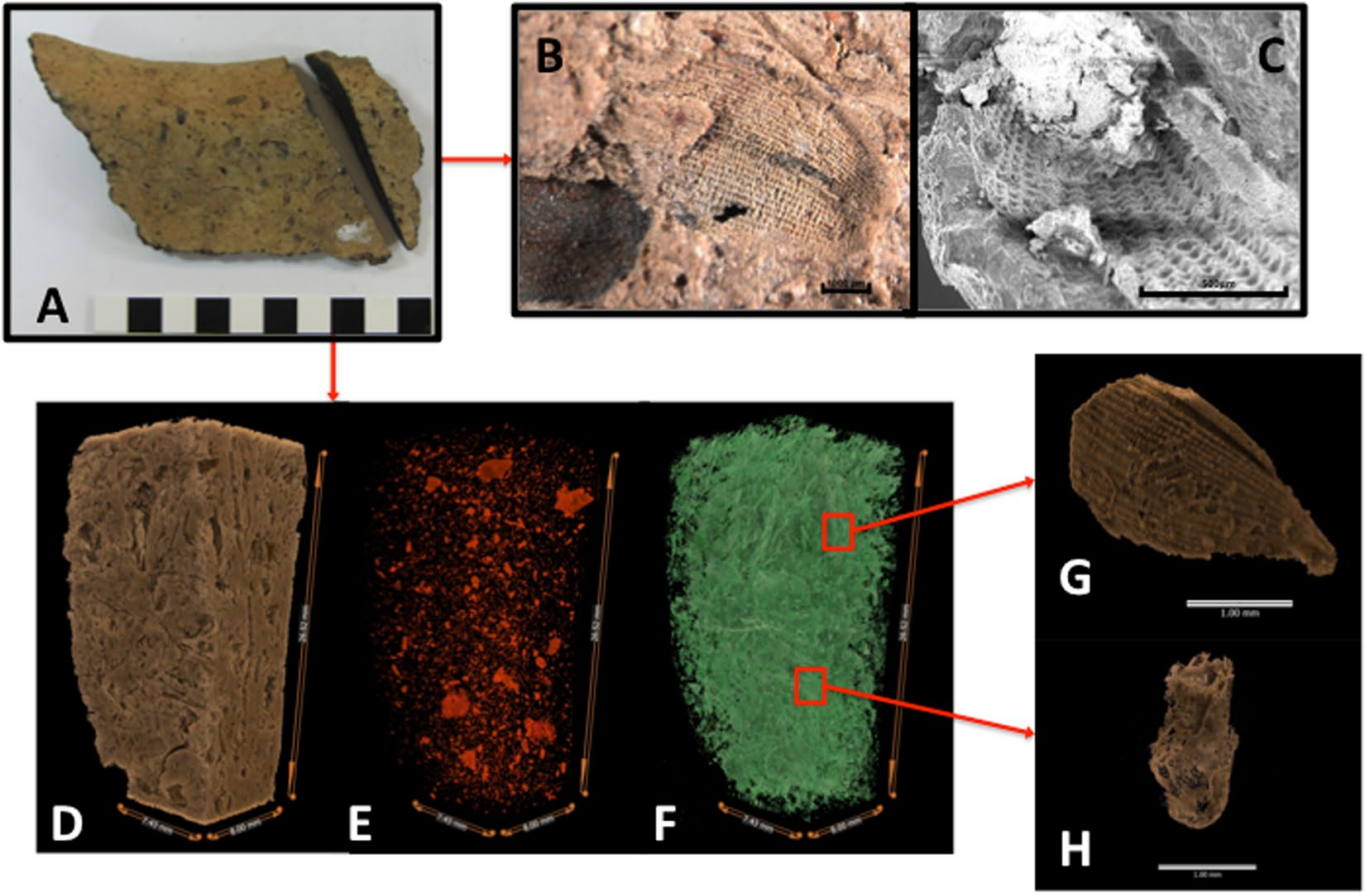
(**A**) – Photograph of Sherd 1 (An Son) showing offcut used for tomographic imaging. (**B**) – Optical microscope image of rice impression on surface of Sherd 1. (**C**) – SEM image of rice impression on exposed cut surface of Sherd 1. (**D**) – Tomograph showing high-density, highly attenuated, clay fraction within offcut of Sherd 1. (**E**) – Tomograph showing mineral fraction within offcut of Sherd 1. (**F**) – Tomograph of low-density organic inclusions within offcut of Sherd 1. (**G**) – Tomograph of targeted rice husk fragment in offcut of Sherd 1, showing distinctive checkboard patterning of epidermis. (**H**) – Tomograph of rice spikelet base in offcut of Sherd 1, identified by positional relationship with husk fragments and recessed, irregular abscission scar. See Animations S1–S5 for detail of Fig. 2D–G.

Here, multiple imaging methods of varying scales and resolution were employed to detect inclusions or impressions within pottery sherds with the intention of identifying rice (*Oryza* spp.) and to positively discriminate between wild and domesticated rice (*Oryza sativa*) (Fig. 2). Sherds were described visually at the macro-scale (Fig. 2A), under optical microscope, SEM, X-ray and microCT. Optical microscopy (Fig. 2B) and SEM (Fig. 2C) provided detailed images of husk impressions within the pottery surface, yet these were not diagnostic of domesticated rice because these types of images are unable to capture the 3D morphological characteristics resulting from human selection, namely the recessed abscission scar^22^. Husk morphologies are insufficient to accurately differentiate potential wild rices growing in the natural environment from domesticated rice cultivated by people. Problematically, most current identifications of putatively domesticated rice in pottery are based on surficial impressions of husks in Southeast Asia^23, 24^ and their domestication status is thereby unsubstantiated.

Sub-samples of each sherd were dissolved and processed to yield phytoliths of domesticated rice. However, no diagnostic rice phytoliths were identified in any sherd. Further, the discrimination of domesticated from wild rice using phytoliths is contentious, although increasingly used in East Asian research^25–27^.

A 1 cm off- cut from each pottery sherd was scanned at the ‘National Laboratory for X-ray Micro Computed Tomography (CTLab)’ based at the Australian National University (ANU) using a HeliScan MicroCT system to yield images at a resolution of 5–8 μm^28, 29^. MicroCT data for each sherd was rendered using Drishti v2.3.2 and Drishti Paint v.2.6 software^30^ to differentiate clay matrix from mineral and organic inclusions (Fig. 2D–F and Supplementary Animations S1–4) and to analyse morphological typography. Following digital isolation of the organic fraction within each sherd, individual organic inclusions of potential rice spikelet bases and husks were targeted for higher resolution processing. The resultant visualisations of individual rice husks (Supplementary Animations S5 and 6) and non-shattering spikelet bases (Supplementary Animations S7–9) were characteristic of domesticated rice in sherds from An Son and Loc Giang. Rice inclusions were not identified in sherds from Rach Nui, which contained mostly low-density non-organic inclusions, possibly reflecting mineral dissolution, and a seed-like inclusion with organic detail (Supplementary Animation S10).

**Figure 3:**
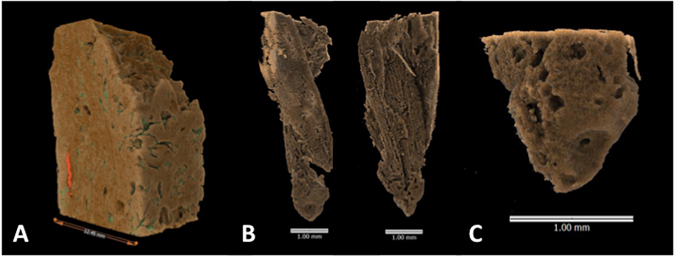
(**A**) – Tomograph of offcut from Sherd 5, Loc Giang, showing organic component in green and position of rice husk (**B**) and attached spikelet base (**C**) in red (Animation S6). (**B**) – Front and side view of tomograph of rice husk and attached spikelet base in offcut of Sherd 5. (**C**) – Closeup of tomograph of spikelet base in Sherd 5, showing irregular, and recessed, abscission scar at base of near complete husk, as well as evidence of rachis pore (Animation S7). Recessed nature of scar shows that force was used to remove seed husk from plant rather than naturally occurring wind dispersal.

**Figure 4:**
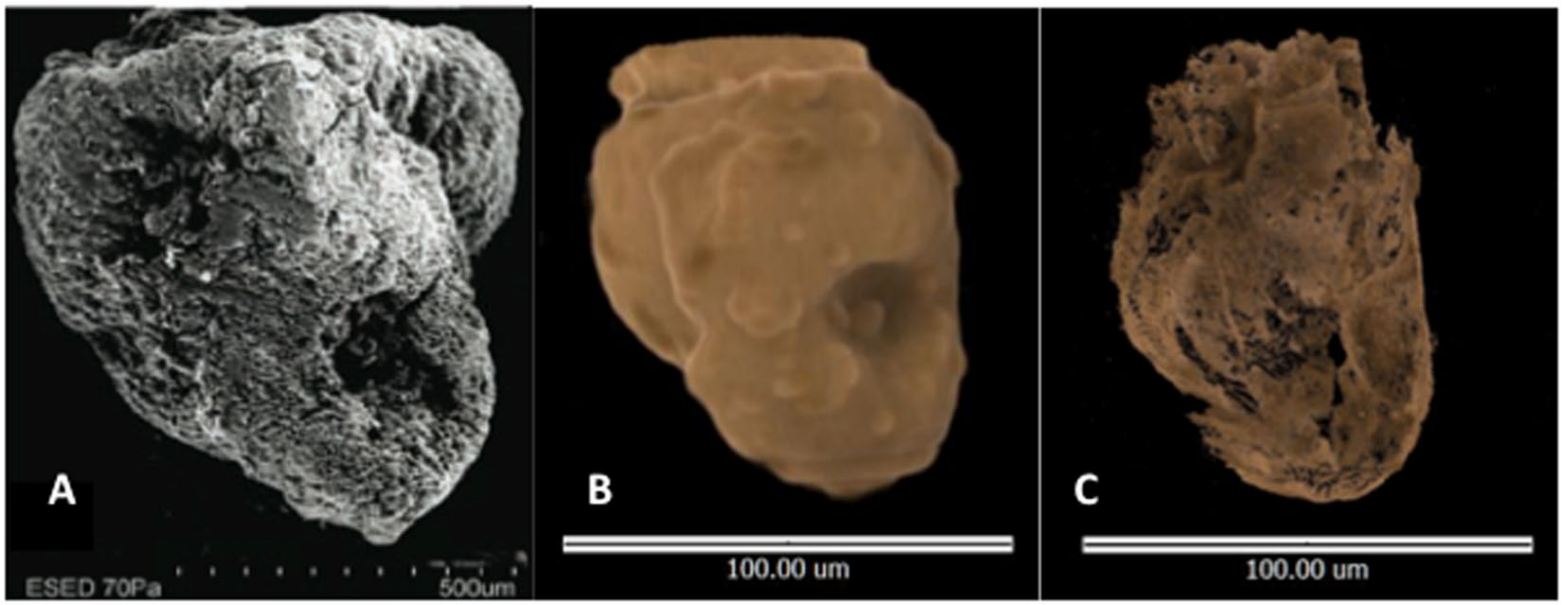
(**A**) – SEM image of domesticated-type rice spikelet base (from Fuller *et al*.^4^ Fig. 3G). (**B**) – Tomographic image of domesticated-type spikelet base in Sherd 3 from Loc Giang (Animation S8). (**C**) – Tomographic image of domesticated-type rice spikelet base in Sherd 1 from An Son (Animation S9).

The distinctive checkerboard patterning of the external surfaces of rice husks was clearly discernible in the optical (Fig. 2B) and SEM images (Fig. 2C), as well as the tomographs, of sherds from An Son and Loc Giang. MicroCT enabled high resolution visualisation of near-complete rice husks (Fig. 2G) and spikelet bases (Fig. 2H) within pottery sherds from these two sites, including a near-complete spikelet base with attached husk from Loc Giang (Fig. 3 & Supplmentary Animation S8). Spikelet base inclusions were compared to SEM reference images of domesticated, wild and immature rice spikelet bases (Fig. 4). All abscission scars on inclusions within pottery corresponded to the domesticated type, primarily due to irregular shape, concavity and size (Supplementary Animations 5–7).

The asynchronies between previous archaeobotanical data and the microCT analyses at these three sites may represent the vagaries of archaeological preservation of plant remains in the wet tropics. However, they may also indicate differences between communities cultivating and those importing domesticated rice. For instance, the absence of domestic rice inclusions within pottery made locally at Rach Nui, yet its reported presence in archaeobotanical assemblages there, may indicate people imported rice for consumption rather than growing it locally^30^. If grown locally, domesticated rice chaff, husks and spikelet bases would be expected to occur as temper within pottery made at the site. Anecdotally, local villagers there today suggest the estuarine environment was too saline to grow rice until recently. However, caution is needed as the limited application of modern tropical archaeobotanical methods in mainland Southeast Asia and the preliminary application of microCT to pottery assemblages precludes more refined interpretation.

In this study, microCT has been applied to positively discriminate domesticated rice inclusions within pottery sherds from two early Neolithic sites in Southeast Asia. The accurate study of rice inclusions and impressions within pottery opens up new possibilities for understanding the spread of domesticated rice from China to Vietnam and Thailand on mainland Southeast Asia, as well as Taiwan, the Philippines and Borneo in Island Southeast Asia. The dispersal of domesticated rice in Southeast Asia has previously been inferred using rice husk impressions in pottery or from phytoliths; although the accuracy of both methods for differentiating domesticated from wild rice is unclear. MicroCT is a reliable diagnostic technique for the discrimination of domesticated rice within pottery sherds and its application may be especially significant in the wet tropics where botanical preservation in archaeological contexts is often poor. The technique is non-destructive, thereby enabling the analysis of crop remains, as well as potentially other materials, within pottery at sites where only a few key sherds have been preserved, or for regions where archaeobotanical methods have not been systematically applied. Further, microCT offers broad potential for tracking the domestication of major crops in other regions, such as cereals and legumes in Africa and Southwest Asia, through the analysis of chaff-tempered pottery, as well as the subsequent dispersal of these crops.

## Electronic supplementary material

Supplementary information.

S1

S2

S3

S4

S5

S6

S7

S8

S9

S10
