# Supplementary material for: MicroCT reveals domesticated rice (Oryza sativa) within pottery sherds from early Neolithic sites (4150–3265 cal BP) in Southeast Asia
Source: Sci Rep. 2017 Aug 7;7:7410. doi: 10.1038/s41598-017-04338-9 (PMC5547045; doi:10.1038/s41598-017-04338-9)
Supplement: Supplementary file 1 — Supplementary information. [file 41598_2017_4338_MOESM1_ESM.doc]

**MicroCT reveals domesticated rice (*Oryza sativa*) within pottery sherds from early Neolithic sites (4150-3265 cal BP) in Southeast Asia**

Aleese Barron**[[1]](#footnote-2)**, Michael Turner**[[2]](#footnote-3)**, Levi BeechingError: Reference source not found, Peter Bellwood^1^, Philip PiperError: Reference source not found, Elle Grono^1^, Rebecca Jones^1^, Marc OxenhamError: Reference source not found, Nguyen Khanh Trung Kien[[3]](#footnote-4), Tim Senden**[[4]](#footnote-5)**, Tim DenhamError: Reference source not found*

**Supplementary Legends**

Animation S1– 3D animation of tomographic data of Sherd 1, An Son, with clay fraction in brown, organic fraction in green and mineral fraction in red

Animation S2 – 3D animation of tomographic data depicting clay fraction of Sherd 1, An Son (Fig. 2D)

Animation S3 - 3D animation of tomographic data depicting mineral fraction of Sherd 1, An Son (Fig. 2E)

Animation S4 - 3D animation of tomographic data depicting organic fraction of Sherd 1, An Son (Fig. 2F)

Animation S5 - 3D animation of tomographic data depicting rice husk inclusion in Sherd 1, An Son (Fig. 2G)

Animation S6 - 3D animation of tomographic data depicting rice spikelet base and attached husk inclusion in Sherd 5, Loc Giang (Fig. 3B)

Animation S7 - 3D animation of tomographic data depicting rice spikelet base inclusion in Sherd 5, Loc Giang (Fig. 3C)

Animation S8 - 3D animation of tomographic data depicting rice spikelet base inclusion in Sherd 3, Loc Giang (Fig. 4B)

Animation S9 – 3D animation of tomographic data depicting rice spikelet base inclusion in Sherd 1, An Son (Fig. 4C)

Animation S10 – 3D animation of tomographic data depicting seed-like inclusion in Sherd 4, Rach Nui

1. School of Archaeology and Anthropology, Australian National University, Canberra, ACT, 2601, Australia. [↑](#footnote-ref-2)
2. ## National Laboratory for X-ray Computed Tomography, Australian National University, Canberra, ACT, 2601, Australia.

   [↑](#footnote-ref-3)
3. Centre for Archaeological Studies, Southern Institute for Social Sciences, Ho Chi Minh City, Vietnam. [↑](#footnote-ref-4)
4. Research School of Physics and Engineering, Australian National University, Canberra, ACT, 2601, Australia.

   *corresponding author [↑](#footnote-ref-5)
